# *In-vivo* Performance of Seven Commercially Available Demineralized Bone Matrix Fiber and Putty Products in a Rat Posterolateral Fusion Model

**DOI:** 10.3389/fsurg.2020.00010

**Published:** 2020-03-20

**Authors:** Nicholas Russell, William R. Walsh, Vedran Lovric, Peter Kim, Jennifer H. Chen, Michael J. Larson, Frank Vizesi

**Affiliations:** ^1^SeaSpine Inc., Carlsbad, CA, United States; ^2^Surgical and Orthopaedic Research Laboratories, Prince of Wales Clinical School, University of New South Wales, Sydney, NSW, Australia; ^3^Ibex Preclinical Research Inc., Logan, UT, United States

**Keywords:** demineralized bone fiber, demineralized bone matrix, bone graft, spinal fusion, carrier, posterolateral spinal fusion, athymic rat

## Abstract

**Introduction:** Demineralized bone matrix (DBM) is a widely used bone graft in spinal fusion. Most commercial DBMs are composed of demineralized bone particles (~125–800 microns) suspended in a carrier that provides improved handling but dilutes the osteoinductive component. DBM fibers (DBF) provide improved osteoconductivity and do not require a carrier. It has been suggested that 100% DBF may offer improved performance over particulate-based DBMs with carrier.

**Study Design:** Seven commercially available DBM products were tested in an athymic rat posterolateral fusion model. There were four 100% DBFs, two DBFs containing a carrier, and one particulate-based DBM containing carrier.

**Objective:** The study objectives were to evaluate the *in vivo* performance: (1) compare fusion rate and fusion maturity of six commercially available DBFs and one particulate-based DBM, and (2) assess the effect of carrier on fusion outcomes for DBFs in a posterolateral fusion model.

**Methods:** The DBF/DBM products evaluated were: Strand^TM^ Family, Propel® DBM Fibers, Vesuvius® Demineralized Fibers, Optium® DBM Putty, Grafton® DBF, Grafton Flex, and DBX® Putty. Single-level posterolateral fusion was performed in 69 athymic rats. Fusion was assessed bilaterally after 4 weeks by manual palpation, radiograph and CT for bridging bone. Fusion mass maturity was assessed with a CT maturity grading scale and by histology. Statistical analysis was performed using Fishers Exact Test for categorical data and Kruskal-Wallis Test for non-parametric data.

**Results:** Strand Family achieved 100% fusion (18/18) by manual palpation, radiographic and CT evaluation, significantly higher than Propel Fibers, Vesuvius Fibers, Optium Putty, and DBX Putty, and not statistically higher than Grafton DBF and Grafton Flex. Strand Family provided the highest fusion maturity, with CT maturity grade of 2.3/3.0 and 89% mature fusion rate. Fusion results suggest a detrimental effect of carrier on fusion performance.

**Conclusions:** There were large variations in fusion performance for seven commercially available DBM products in an established preclinical fusion model. There were even significant differences between different 100% DBF products, suggesting that composition alone does not guarantee *in vivo* performance. In the absence of definitive clinical evidence, surgeons should carefully consider available data in valid animal models when selecting demineralized allograft options.

## Introduction

Spinal arthrodesis is a widely performed surgical procedure used to treat numerous spinal pathologies. Autologous bone harvested from the iliac crest is often considered the gold-standard graft for spinal fusion procedures. However, supply is often limited, and its harvest is associated with increased surgery time, blood loss and risk of infection. Additionally, chronic donor site pain or morbidity has been reported in 8–26% of patients (1–3). There continues to be demand for alternative bone graft materials that can replace autograft bone harvested from the iliac crest and/or augment local autograft bone for spinal fusion procedures.

Demineralized Bone Matrix (DBM) is a type of bone graft alternative that is processed from human allograft bone. DBM is processed by removing the mineral component of bone with acid, leaving behind the extracellular matrix composed of collagen and non-collagenous proteins, including the endogenous growth factors. The presence of these endogenous growth factors, particularly BMPs, imparts osteoinductive properties, while the geometry of the collagen matrix has the potential to impart varying degrees of osteoconductivity to the graft (4). DBM has become one of the most widely used bone graft alternatives in spinal fusion surgery. DBM possesses several qualities that make it an attractive graft option. It is readily available, cost-effective, and requires little or no preparation.

Clinical studies have reported the safety and efficacy of DBM as a bone graft extender in lumbar spine fusion (5–8). One study in 120 patients undergoing 1–2 level instrumented posterolateral fusion (PLF) used a DBM gel (Grafton®) mixed with local autograft bone implanted on one side, compared to iliac crest autograft implanted on the contralateral side in the same patient (9). Fusion rates at 24 months were 52% for the DBM composite group and 54% for the iliac crest group. Another study in 59 patients undergoing 1–2 level instrumented PLF evaluated a DBM putty (Accell Connexus®) mixed with iliac crest or local autograft, compared to iliac crest or local autograft alone (10). Radiographic fusion rates after 12 months were similar between the two groups, with 70% fusion for the DBM composite group and 77% for autograft alone. Other studies report similar findings of comparable fusion rates and clinical outcomes for local autograft-DBM composites compared to iliac crest alone (11, 12). Overall, the clinical evidence supports DBM as an effective bone extender when used to augment a smaller quantity of autograft, offering similar clinical performance to autograft in posterolateral spinal fusion.

There are currently numerous DBM products commercially available for use in spinal fusion surgeries. These are available in different forms including powders, putties, gels, pre-filled syringes, pouches, strips, fibers, and others. However, the bone-forming capacity of these products has been reported to vary considerably (13–18). This may be attributed to several factors. Since commercially available DBMs are processed by different manufacturers or tissue banks, there is variability in its production from allograft bone. Sources of variability include quality of the donor bone, bone geometry, demineralization methods, sterilization method, and use of a carrier, which can result in inconsistent biologic responses (19).

The purpose of the carrier medium in DBM products is to improve the handling characteristics, but its use comes at the expense of active DBM component, diluting the osteoinductive performance of the product. Traditionally, the active DBM component extracted from bone is a fine particulate powder that is difficult to handle and deliver in surgery. The addition of inert, biocompatible carriers is intended to turn the DBM powder into a putty or paste to make it easier to localize to the fusion site. Examples of DBM carriers used in commercially available products include glycerol, hyaluronic acid, poloxamer reverse phase medium, gelatin, and others. Unfortunately, for many commercial DBM putty-type products, a major portion of the final DBM complex is the carrier (e.g., up to ~85% carrier and 15% active DBM), which decreases the amount of active DBM component that can be incorporated (6). Furthermore, upon dissolution of the carrier material, the graft volume may decrease and leave voids in the implantation site. Due to the drawbacks of carrier materials, there has been an increase in demand for DBM products composed of 100% active DBM.

Demineralized bone fibers (DBF) are a formulation of demineralized bone matrix that can provide favorable handling without the need of a carrier. To manufacture DBF, allograft bone is demineralized in the form of long fibers or ribbons, rather than fine particulate, so the resulting DBF product is cohesive on its own. However, different fiber geometry and product configurations can result in variable handling and biologic properties. One commercially available DBF (OsteoStrand™ Plus) is composed of 100% DBM in the form of long fibers that exhibit controlled expansion after implantation to maximize implant fill. In contrast, there are other commercial preparations of DBF that do still include a carrier and may be compressed into different shapes (e.g., strips).

In addition to improved handling as well as being osteoinductive, DBF has also demonstrated an osteoconductive advantage over DBM particulate in a rabbit model of posterolateral spine fusion (4). In a study by Martin et al. (4), two fiber-based formulations of DBM (Grafton Flex and Putty) were compared to a particle-based formulation (Grafton gel). All three DBMs had the same osteoinductivity, but the fiber-based DBMs demonstrated a higher fusion rate at 92% compared to the particle-based DBM fusion rate of 58%. When osteoinductivity was removed from the DBMs using guanidine extraction to remove the inductive protein pool, leaving behind only osteoconductive properties, the fiber-based DBM fusion rate decreased to 36%, whereas the particle-based DBM dropped to 0%. This suggests that the fiber-based versions of DBM provide greater osteoconductivity than particulate-based DBMs to aid in new bone formation.

There is an increasing variety of DBF products being released commercially, with variable fiber geometries and product configurations. While there may be benefits to the fiber-based DBM format, there is a need for greater understanding of the factors affecting bone-forming capacity and fusion performance of DBFs. The main objective of this study was to compare the fusion performance of different commercially available DBM fiber and putty products in a single-level posterolateral fusion model. The second objective of this study was to assess the effect of carrier on *in vivo* fusion outcomes of DBF in a posterolateral fusion model.

## Materials and Methods

### Study Design

Seven different commercially available demineralized bone fiber and putty products were tested in a single-level athymic rat posterolateral fusion model (13–18). Table 1 summarizes the product information for each DBM tested in this study. Four of the groups are 100% demineralized bone fibers: Strand™ Family DBM Fibers (SeaSpine, Carlsbad, CA), Propel® DBM Fibers (NuVasive, San Diego, CA), Vesuvius^TM^ Demineralized Fibers (LifeNet, Virginia Beach, VA), and Grafton® DBM DBF (Osteotech, Eatontown, NJ). Two of the DBF products contain glycerol carrier: Optium® DBM Putty (Lifenet, Virginia Beach, VA, also distributed as Vesuvius DBM putty), and Grafton® Flex (OsteoTech, Eatontown, NJ). A traditional particle-based DBM putty containing sodium hyaluronate carrier: DBX® Putty (MTF, Edison, NJ) was included as a control group. DBF and DBM products were selected for being in current widespread clinical use and/or for being manufactured by different AATB-accredited musculoskeletal tissue banks in the United States. Furthermore, two of the DBF product families were selected to enable the comparison of fibers with or without carrier: the Vesuvius family and Grafton family, which each have one product formulation with glycerol carrier (Vesuvius/Optium Putty and Grafton Flex, respectively) and another product formulation that is 100% DBF (Vesuvius Demineralized Fibers and Grafton DBF, respectively).

**Table 1:** DBM Fiber and putty products tested.

**Product name**	**Distributor/manufacturer**	**DBM format**	**Carrier**	**Product composition by weight (%)**		**DBM dry weight (g/cc)**	**Sample size**
				**DBM**	**Carrier**		***N***
Strand™ Family	SeaSpine	Fibers	N/A	100	0	0.302	18
Propel® DBM Fibers	NuVasive (AlloSource)	Fibers	N/A	100	0	*	9
Vesuvius™ Demineralized Fibers	K2M (LifeNet)	Fibers	N/A	100	0	0.164	9
Optium® / Vesuvius DBM Putty	LifeNet/K2M (LifeNet)	Fibers	Glycerol	18	82	0.304	8
Grafton® DBM DBF	Medtronic (OsteoTech)	Fibers	N/A	100	0	0.209	9
Grafton Flex	Medtronic (OsteoTech)	Fibers	Glycerol	43	57	0.353	8
DBX Putty®	DePuy Synthes (MTF)	Particles	Sodium hyaluronate	28	72	0.339	8

^*^Not obtained due to product being packaged wet in saline.

All DBM products were obtained in sterile, factory-sealed packaging for use in humans with at least 6 months shelf life before expiration. Multiple lots (1–3 lots) were obtained for each product. All groups were assessed using the same *in vitro* characterization, *in vivo* implantations, and fusion assessments at 4 weeks post-implantation.

### Surgery and Fusion Assessment

Sixty-nine mature male athymic rats (10–11 weeks) were used following ethical approval. A single-level posterolateral fusion procedure was performed between the L4–L5 vertebra. The transverse processes were exposed by paramedian incision and the dorsal surfaces were gently decorticated with a motorized burr. An aliquot of graft material equal to 0.3 cm3 was placed bilaterally in the prepared posterolateral gutters bridging the decorticated transverse processes for a total of 0.6 cm3 of graft material for each animal. The Strand Family, Propel Fibers, Vesuvius Fibers, Optium Putty, and Grafton DBF, and DBX Putty implants were prepared by packing 0.3 cm3 of graft material into a 1cc open bore syringe for delivery to the surgical site. Grafton Flex was cut into rectangular strips equaling 0.3 cm3 by volume. Strand family, Vesuvius Fibers, Grafton DBF DBM, and Grafton Flex were hydrated with sterile saline prior to implantation. After implantation was complete, all wounds were closed with suture in two layers and an anterior-posterior radiograph of the lumbar spine was taken. All rats were euthanized 4 weeks post-operatively via CO_2_ overdose, and the lumbar spines were harvested en bloc for analysis.

Immediately after harvest, explanted lumbar spines were manually tested for intersegmental motion by two independent trained observers blinded to treatment groups. Any motion detected between the facets or transverse processes of L4 and L5 by manual palpation was considered a failure of fusion. The absence of motion (both right and left) was considered successful fusion. Anterior-posterior Faxitron radiographs (Faxitron, Wheeling, IL) and digital plates (AGFA CR MD4.0 Cassette) were taken of each spine and evaluated in a blinded fashion by 2 independent observers. Fusion was determined by radiographic evidence of bone bridging the transverse processes, with left and right fusion masses evaluated independently.

### Qualitative Fusion Maturity μCT Grading Scale

**Figure 1:**
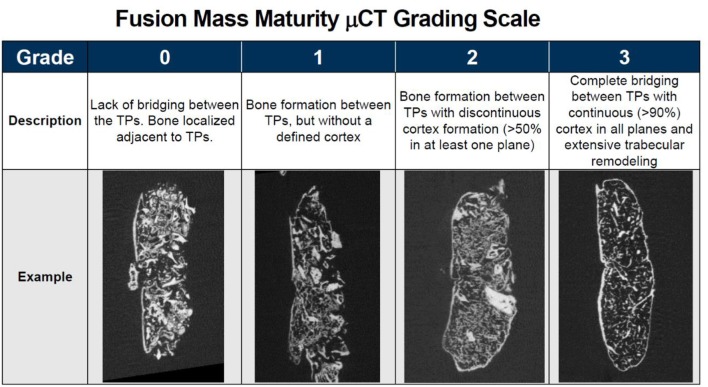
Qualitative fusion mass maturity μCT grading scale with description and representative μCT image of each grade.

Microcomputed tomography (μCT) (Siemens Medical Solutions, Knoxville, Tennessee) scanning was performed on all animals to obtain high resolution radiographic images of the spinal fusions in three planes. Image analysis software, Inveon Research Workplace [IRW] (Siemens Medical Solutions, Knoxville, Tennessee) was used to reconstruct the μCT image data and evaluate the fusions between the treated levels in the coronal and sagittal planes. Fusion was assessed using a qualitative fusion maturity grading scale to score each fusion mass on a scale of 0 to 3 (Figure 1). Grade 0 corresponds to an incomplete or lack of bridging bone spanning the transverse processes and is considered not fused. Grade 1 demonstrates continuous bone formation spanning the transverse processes, but without a defined cortex, and is considered an immature fusion. Grade 2 demonstrates bone formation between transverse processes with discontinuous cortex formation (cortex surrounding >50% of fusion mass in at least one plane). It is considered fused and progressing toward mature fusion. Grade 3 demonstrates complete bridging between transverse processes with continuous (>90%) cortex formation in all planes and extensive trabecular remodeling. It is considered a mature fusion.

The spines were fixed in 10% phosphate buffered formalin and processed for routine Hematoxylin and Eosin (H&E) decalcified in 10% formic acid for paraffin histology. Sagittal histology sections from each side of the fusion were qualitatively assessed to evaluate the maturity of the fusion in each group. Graders were blinded to the treatment group and the results of fusion evaluation from other endpoints.

### *In vitro* Product Characterization

Product characteristics measured were overall product composition (% DBM, % carrier) and DBM content (dry weight of DBM component). A portion of samples from each lot was set aside for *in vitro* analysis. Samples were prepared according to manufacturer's instructions and weighed before and after rinsing out the carrier (if applicable), and lyophilization.

### Statistical Analysis

Statistical analysis was performed using Fishers Exact Test for categorical data and Kruskal-Wallis ANOVA for non-parametric data. A *p*-value < 0.05 was considered statistically significant. Linear regression was used to assess the relationship between fusion rate and percentage composition of carrier, for the carrier-containing products.

## Results

**Figure 2:**
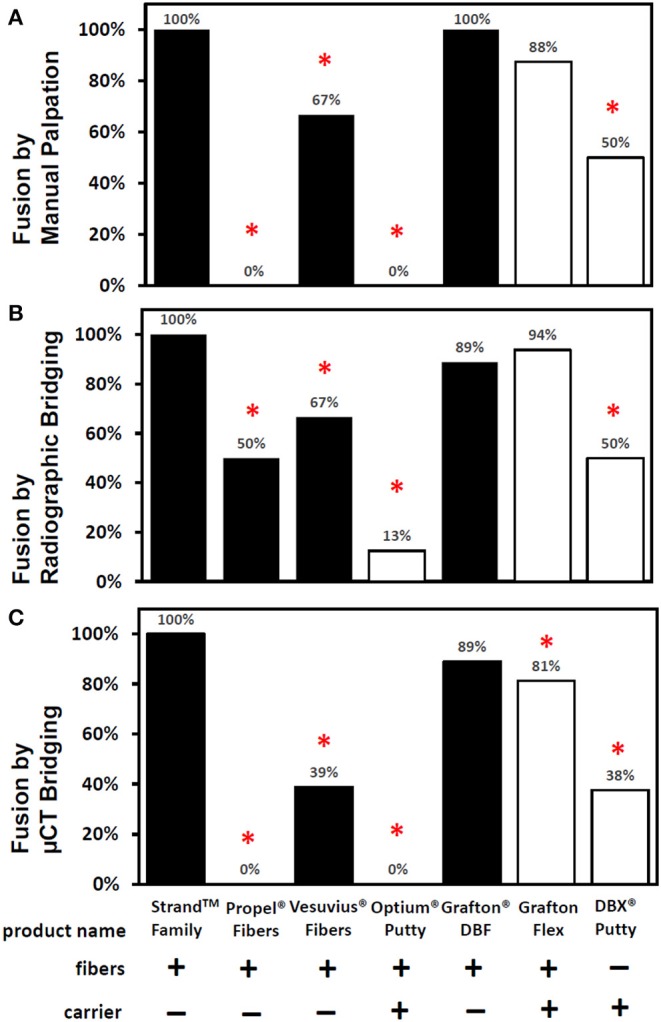
Fusion rates as determined by **(A)** manual palpation **(B)** radiographic bridging and **(C)** μCT bridging bone. *Denotes a significant difference compared to the Strand Family group at *p* < 0.05.

The *in vivo* phase of the study was uneventful with all animals recovering well following surgery and no adverse reactions noted. The results of the fusion assessments from manual palpation, radiography and μCT are presented in Figure 2 and Table 2. All study endpoints were closely concordant in terms of fusion rates. Strand Family demonstrated a fusion rate of 100% (9/9), significantly higher than the 0% (0/8 fused by manual palpation) for Propel Fibers, 67% (6/9) for Vesuvius Fibers, 0% (0/8) for Optium Putty, and 50% (4/8) for DBX Putty (*p* < 0.05). Strand Family fusion rates were not statistically higher than Grafton DBF (100% by manual palpation, 89% by radiograph assessment) or Grafton Flex (88% by manual palpation, 94% by radiograph).

**Table 2:** Fusion rates assessed by manual palpation, X-ray, and CT bridging bone.

**Group**	**MP fusion**	**X-ray bridging**	**CT bridging**
Strand™ Family	100%	100%	100%
Propel® DBM Fibers	0%^*^	50%^*^	0%^*^
Vesuvius™ Demineralized Fibers	67%^*^	67%^*^	39%^*^
Optium® DBM Putty	0%^*^	13%^*^	0%^*^
Grafton® DBM DBF	100%	89%	89%
Grafton Flex	88%	94%	81%^*^
DBX Putty®	50%^*^	50%^*^	38%^*^

* *Statistically significant vs. Strand Family, p < 0.05*.

**Figure 3:**
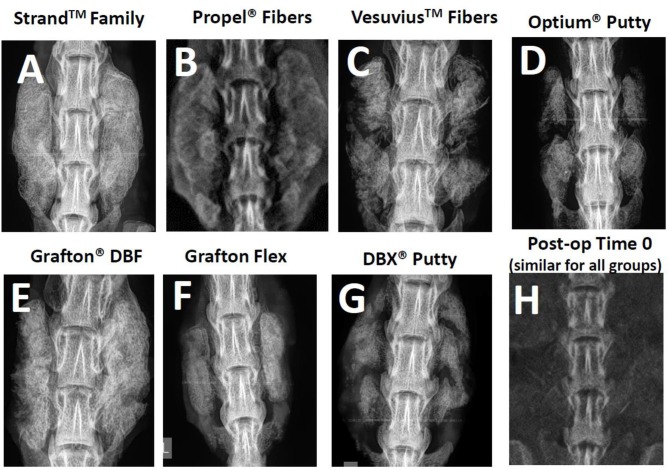
Representative radiographs of each group at 4-weeks post-op **(A–G)**, and a representative time 0 radiograph taken immediately post-implantation, which was similar for all groups **(H)**.

**Figure 4:**
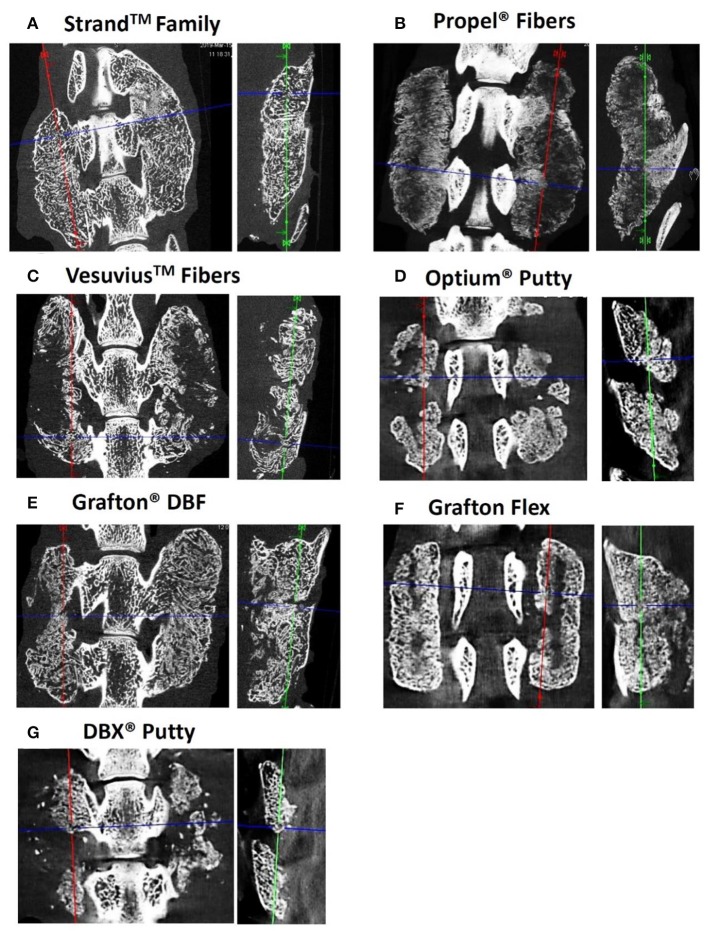
Representative μCTs of each group at 4 weeks demonstrating the differences in bone formation and fusion maturity between groups. Strand Family **(A)** and Grafton DBF **(E)** had large bilateral fusions bridging the transverse processes (TPs) with trabecular remodeling and the presence of a defined cortex; Vesuvius Fibers **(C)** and Grafton Flex **(F)** had centralized voids in the fusion masses; Both putties **(D,G)** were characterized by bone formation localized to the TPs with minimal bridging bone; Propel Fibers **(B)** had soft radiolucent fusion masses with no defined mineralization bridging the TPs.

Radiographic and μCT imaging illustrated substantial differences in the amount and quality of bone formation in the fusion masses between the groups (Figures 3, 4). Faxitron radiographs and μCT reconstructions of the Strand Family and Grafton DBF groups displayed large bilateral fusions with contiguous bone masses bridging between the L4-L5 transverse processes in all animals (Figures 3A,E, 4A,E). In contrast, bone formation for Vesuvius Fibers, Optium Putty, and DBX Putty groups was typically localized around the host transverse processes and in disparate islands between the transverse processes, (Figures 3C,D,G, 4C,D,G). Likewise, although bone formation was evident in the Propel Fibers and Grafton Flex groups, the fusion mass often possessed a large central void (Figures 4D,F).

**Figure 5:**
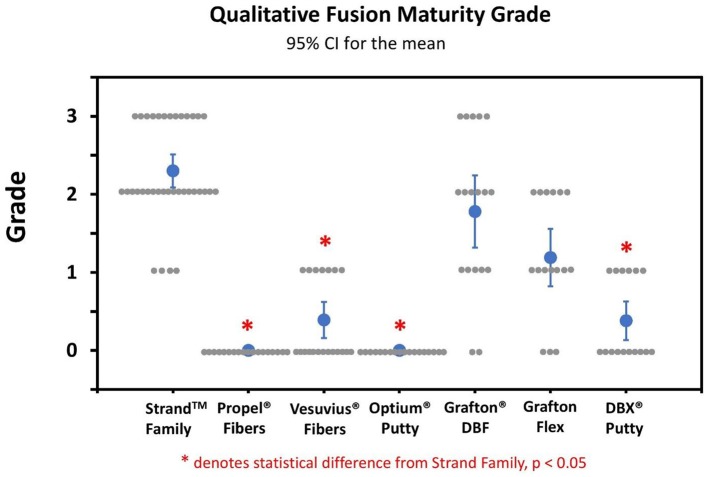
Qualitative fusion μCT maturity grade scatter plot of all groups. Blue dots indicate mean ± SD, gray dots indicate individual sample scores, *Indicates statistical difference from Strand Family, *p* < 0.05.

Qualitative grading of fusion revealed significant differences in fusion bone maturity even among groups with similar fusion rates (Figure 5). Strand Family achieved a μCT fusion maturity grade of 2.3 out of 3.0, significantly higher than Propel Fibers, Vesuvius Fibers, Optium Putty, and DBX Putty (Figure 5). Although Grafton DBF and Grafton Flex had lower maturity grades of 1.78 and 1.19 compared to Strand, the differences did not reach statistical significance. Correspondingly, Strand Family had an 89% mature fusion rate, where mature fusion is defined as Grade 2 or Grade 3 on the μCT fusion maturity grading scale (Table 3). Strand Family fusion maturity was 89%, compared to 61% for Grafton DBF and 38% for Grafton Flex, but differences were not statistically significant. Strand Family was statistically higher than Propel Fibers, Vesuvius Fibers, Optium Putty, and DBX Putty which all had 0% mature fusions.

**Table 3:** Fusion mass maturity assessed by μCT.

**Group**	**CT maturity grade (0–3)**	**Mature fusion mass by CT (%)**
Strand™ Family	2.3 ± 0.6	89%
Propel® DBM Fibers	0 ± 0^*^	0%^*^
Vesuvius™ Fibers	0.39 ± 0.5^*^	0%^*^
Optium®/Vesuvius DBM Putty	0 ± 0^*^	0%^*^
Grafton® DBM DBF	1.78 ± 1.0	61%
Grafton® Flex	1.19 ± 0.8	38%
DBX Putty®	0.38 ± 0.5^*^	0%^*^

* *Statistically significant vs. Strand Family, p < 0.05*.

**Figure 6:**
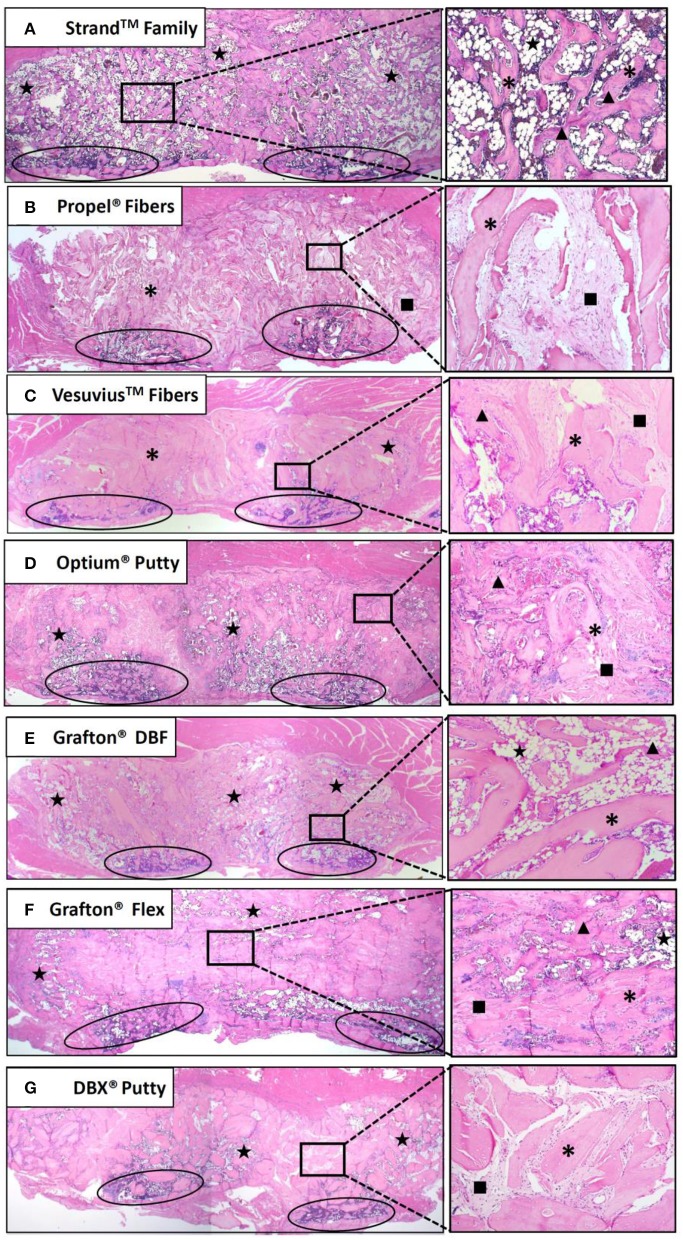
Representative H&E histology images of fusion masses in each group. Strand Family **(A)** and Grafton DBF **(E)** had DBM fibers incorporated into bone marrow-filled bridging bone. Propel Fibers **(B)**, Vesuvius Fibers **(C)**, Optium Putty **(D)**, and DBX Putty **(G)** had residual DBM surrounded by fibrous tissue with little bone remodeling. Grafton Flex **(F)** had bone formation at the periphery of the fusion mass and fibrous tissue in the center. Ovals indicate transverse processes, star indicates marrow, *indicates residual DBM, ▴ indicates new bone, ■ indicates fibrous tissue. Image magnification 1.25x objective (left) and 10x objective (right).

Histological evaluation supported the radiographic observations in terms of both bridging bone from transverse process to transverse process and the formation of new cortex. The Strand Family specimens demonstrated clear trabeculae bridging from one transverse process to the next, incorporating the DBM fibers within a bone marrow remodeled fusion mass (Figure 6A). There was little interposed fibrous tissue in these specimens. In contrast, the Propel Fibers, Vesuvius Fibers, Optium Putty, and DBX Putty groups showed bone formation adjacent to the transverse processes, but with predominantly fibrous tissue interposed between them (Figures 6B–G). In these four groups the DBM material in the center of the fusion mass was infiltrated with hypocellular fibrous tissue, with minimal new woven bone formation. The DBM particulate in the DBX fusions were clearly visible with minimal resorption and remodeling noted (Figure 6G). The Grafton DBF group demonstrated trabecular bone and marrow spaces like the Strand Family group, but also had long fibers of residual DBM within the fusion mass (Figure 6E). The Grafton Flex group was characterized by bone formation and small bone marrow cavities at the periphery of the implanted material (Figure 6F). In the center, there was fibrous tissue infiltration with no trabecular remodeling, and a general hypocellular characteristic, resulting in a “hollow” fusion mass. There was no evidence of inflammatory cell population for all groups.

The *in vitro* product characterization revealed significant differences in product composition (% DBM vs. % carrier) and DBM content by dry weight (Table 1). For the carrier-containing groups, Optium Putty had the lowest % DBM content at 18% DBM (82% carrier), followed by DBX Putty at 28% DBM (72% carrier), and Grafton Flex at 43% DBM (57% carrier). For products containing carrier, there was a linear inverse correlation between percentage carrier in the product and the fusion rate (*R*^2^ = 0.99). DBM content was slightly higher in the carrier-containing DBMs with an average DBM content of 0.33 g/cc, compared to 100% DBF groups which averaged 0.24 g DBM/cc, but the difference was not statistically significant.

## Discussion

There were large variations in fusion performance for seven commercially available DBF and DBM products in an established preclinical fusion model. These data agree well with previous reports using the athymic rat spinal fusion model (13–18, 20–24). Among different 100% DBF products with no carrier present, there were significant differences in fusion outcomes, suggesting that composition alone does not guarantee performance. For the carrier-containing DBF/DBM groups, the results suggest a detrimental effect of carrier on fusion outcomes. The current study did not investigate whether this detrimental effect was due to direct adverse effects of the carrier material or to indirect effects of displacing or diluting the active DBM component.

There were several limitations of this study. A relatively small number of donor lots was tested for each product due to lack of product availability and a single time point at 4 weeks. Furthermore, because the control group of the particle-based DBM had carrier, we were unable to separate the benefit of DBM fibers over particulate from the effect of having no carrier. In this study the DBM and DBF grafts were used alone, whereas in the clinical setting these bone grafts are often mixed with autograft and/or bone marrow aspirate.

The athymic rat spinal fusion model is well-characterized and has been used extensively to test human DBM products (13–24). In this model, DBM and DBF grafts can be tested in an unaltered “off-the-shelf” form, as would be available for use in human spine surgery. In addition, the athymic rat posterolateral fusion is a challenging model that requires the graft to possess both significant osteoinductive and osteoconductive abilities to induce a solid arthrodesis (4). Because of this challenging environment, the reported fusion rates for DBM products tested in this model at 4 weeks vary considerably, which makes it a robust model to discern differences in product performance. Previous studies have evaluated fusion performance of commercial DBM putties and/or gels composed of different carriers and reported fusion rates of 0–100% in the same preclinical fusion model as the current study (13–18, 20–24). In studies by the Wang group (14, 15), substantial variability in performance was measured between different DBM products from different manufacturers, while Bae et al. (17), further showed substantial variability between various lots of the same DBM material. Indeed, in the current study, the full range of fusion rates from 0 to 100% was observed for different groups, as well as corresponding differences in the quality and maturity of bone formation among different DBF and DBM products.

Fusion endpoint evaluated by multiple techniques in the current study were generally concordant, with a tendency for higher fusion rates assessed by manual palpation and for lower fusion rates assessed by μCT. This is consistent with previous studies demonstrating that biomechanically solid fusion occurs prior to radiographic appearance of solid fusion (25). Histologically, the fusion mass initially consists predominantly of woven bone, which provides biomechanical stability, and then becomes gradually trabeculated as the bone remodels. This was evident in the current study, where different groups had distinct qualitative differences in histological appearance. For example, although Grafton Flex had a high fusion rate of 88% by manual palpation, μCT imaging and histology of the fusion mass revealed the presence of a hypocellular central void filled with fibrous tissue. In contrast, in the Strand Family and Grafton DBF groups, the fusion bone was a solid mass with extensive trabecular remodeling surrounded by a cortex. This indicated that different DBF or DBM grafts which were both evaluated as solidly fused could be at different stages of bone maturity. Hence, it is valuable to develop more stringent criteria to distinguish fusion performance beyond the initial phase of biomechanical stability.

A novel μCT fusion maturity grading scale was useful and complementary with histological analysis for evaluating the fusion bone quality and maturity in the current study. The μCT grading scale is distinguished by its ability to semi-quantitatively assess the trabecular remodeling and cortex encompassing the entire three-dimensional fusion mass. However, histological analysis is still necessary to evaluate the cellular and tissue remodeling response in the fusion mass, including any presence of marrow elements, fibrous infiltration, and residual implant material. The distribution and position of these components relative to the host transverse processes and to the newly forming fusion mass bone can help reveal the mechanisms and quality of bone formation. For example, Strand Family displayed newly formed trabecular bone with marrow spaces throughout the fusion mass, including the center of the fusion mass. This is indicative of an osteoinductive response which has resulted in new bone formation and functional bony remodeling. In contrast, Propel Fibers, Vesuvius Fibers, Optium Putty, and DBX Putty groups demonstrated central regions consisting of residual fibers surrounded by fibrous infiltration. This reflects the challenging nature of the posterolateral fusion environment, which requires both strong osteoinductive signal and a favorable osteoconductive scaffold to induce bone formation away from the adjacent host bone. Time is another important variable to consider in the overall paradigm of bone healing.

The variability in fusion performance between 100% DBF products is likely driven by variations in allograft bone processing conditions between different tissue banks. Indeed, an anonymous survey of four AATB accredited tissue banks reported four completely different chemical processing methods were used to ensure the sterility of demineralized bone products (26). While the effect of these different chemical processing methods has not been investigated, it demonstrates the inherent variability in manufacturing of DBM products across different tissue banks. Furthermore, other studies have reported variability in DBM performance arising from processing variables such as differences in donor quality (17, 27, 28), demineralization (29), storage (30, 31), and sterilization procedures (32–34). Qiu et al. (31) investigated the effect of e-beam sterilization on DBM in a hydrous (wet) state compared to an anhydrous (dry) state and reported a 22% reduction in osteoinductivity in the wet DBM. Similarly, Han et al. (30) reported time- and temperature-dependent reductions in osteoinductivity for DBM stored in a wet state for as little as 5 weeks. While these factors were not directly investigated in this study, the results of the products containing moisture (Propel Fibers, Optium Putty, and DBX Putty) in the current study had inferior fusion performance.

Results from preclinical models may not necessarily translate directly to clinical outcomes. However, the relative performance between materials may be of importance for clinical decision making. Further preclinical investigation utilizing well-designed studies that isolate processing variables and product characteristics are required to provide clinicians with the tools to make informed decisions regarding their DBM graft selection. In the absence of definitive clinical evidence, surgeons should carefully consider available data in valid animal models when selecting their demineralized bone-based products.

## Data Availability Statement

The datasets generated for this study are available on request to the corresponding author.

## Ethics Statement

The animal study was reviewed and approved by UNSW Animal Care and Ethics Committee.

## Author Contributions

NR, WW, VL, PK, ML, and FV contributed conception and design of the study. FV, WW, and JC performed the statistical analysis. JC wrote the first draft of the manuscript. NR, WW, and FV wrote sections of the manuscript. All authors contributed to manuscript revision, read and approved the submitted version.

### Conflict of Interest

NR, PK, JC, and FV are employed by SeaSpine, Inc. ML is employed by Ibex Preclinical Research, Inc. WW is on the editorial board for this journal. The remaining author declares that the research was conducted in the absence of any commercial or financial relationships that could be construed as a potential conflict of interest.
